# Surgery with minimal bleeding

**DOI:** 10.1002/ags3.12774

**Published:** 2024-01-11

**Authors:** Daisuke Ichikawa

**Affiliations:** ^1^ First Department of Surgery, Faculty of Medicine University of Yamanashi Yamanashi Japan

Several clinical studies have been conducted to establish improved treatment options for patients with various cancers. There are, however, various aspects of better treatment, and it is important not only to provide better oncological outcomes but also to maintain the patient's quality of life after treatment.

In this issue, Toriumi et al.^1^ reported a supplemental analysis of the randomized controlled JCOG1001 trial, which primarily investigated the superiority of bursectomy over conventional omentectomy in patients with cT3‐4 advanced gastric cancer. They examined the efficacy of an anti‐adhesion membrane in preventing postoperative small‐bowel obstruction (SBO) in patients undergoing open gastrectomy. SBO is a major complication in surgically treated patients with cancer and frequently affects them during long‐term postoperative periods, even after complete healing. The authors demonstrated that the anti‐adhesion membrane did not decrease the occurrence of SBO after surgery. Meanwhile, it was found that a large amount of blood loss was independently associated with SBO development in the multivariate analysis. Based on these findings, surgeons should strive to minimize intraoperative bleeding rather than rely on anti‐adhesion membranes to prevent SBO.

Various reports and discussions have been published regarding the prognostic impact of intraoperative blood loss (IBL) and its effects on intra‐abdominal adhesions. Several studies have demonstrated that IBL adversely affects the prognostic outcome of patients with various cancers, including gastric cancer, after curative resection. Although some studies have failed to identify this negative effect, various studies have set varying thresholds to analyze the clinical impact of IBL, which can potentially lead to varying interpretations. Comprehensive studies, such as meta‐analyses and systematic reviews, have mostly concluded that large amount of IBL certainly has some negative prognostic effects.^2^ Recently, randomized controlled trials have been considered the most important for elucidating the clinical significance of various medical interventions; however, it is ethically impossible to compare the clinical outcomes between large and small IBL groups. Therefore, supplemental and/or exploratory analyses of well‐designed RCTs may be most appropriate for assessing the clinical significance of IBL. Misawa et al.^3^ analyzed the prognostic impact of IBL in the same cohort of JCOG1001 trial described above and clearly demonstrated that an intraoperative blood loss of ≥200 mL was an independent worse prognostic factor in patients after curative gastrectomy for cT3/4a gastric cancer.

Moreover, several studies have reported a relationship between IBL and the recurrence type. Kamei et al. (doi: 10.1007/s00268‐009‐9979‐4.) reported that IBL is a critical risk factor for peritoneal recurrence, but not for nodal and hematogenous recurrences, in patients with gastric cancer. Further, Arita et al. (doi: 10.1245/s10434‐014‐4060‐4.) reported that patients with advanced gastric cancer accompanied by a large amount of intraoperative hemorrhage are more likely to develop peritoneal recurrence and proved that plasma increases the ability of cancer cells and mesothelial cells to adhere to each other. Among other blood components, platelets are known to play a role in various cancers, especially in hematogenous metastases. Saito et al.^4^ investigated the role of platelets in gastric cancer and found that platelets formed complexes with gastric cancer cells in co‐culture and subsequently enhanced their malignant behavior, such as adherence to mesothelial cells and invasion ability, through direct contact. These findings suggest that IBL promotes peritoneal recurrence in the presence of free cancer cells in the abdominal cavity. Nakayama et al.^5^ reported the addition of platelets along with gastric cancer cells resulted in a significant increase in peritoneal dissemination in a mouse model.

The amount of IBL in gastric cancer surgery appears to have decreased owing to the development of various hemostatic devices and the increasing popularity of laparoscopic surgery. Laparoscopic surgery has recently been recognized as the standard treatment for both early and advanced gastric cancer; therefore, the proportion of less invasive surgeries is expected to increase in the future. However, we must once again confirm that clinical trials verifying the validity of laparoscopic gastrectomy were carried out by laparoscopic experts and that the amount of IBL was significantly different among each clinical trial, including in the laparoscopic gastrectomy group (30–152.4 mL). Therefore, rather than merely settling for performing laparoscopic surgery, it is desirable to perform surgery with the aim of minimizing IBL as much as possible, by adopting a comprehensive surgical approach that takes into account both macroscopic and microscopic perspective, while considering the experimental findings mentioned earlier.

Apart from this article, this issue features 15 other original articles and two reviews aimed at improving the therapeutic outcomes of patients with gastrointestinal cancers. These studies, irrespective of their prospective or retrospective nature, were conducted based on the understanding and cooperation of numerous patients. We must engage in a once‐in‐a‐lifetime surgical treatment for each patient by expressing our appreciation for the participants of previous studies.

## FUNDING INFORMATION

This editorial did not receive any specific grant from funding agencies in the public, commercial, or nonprofit organizations.

## CONFLICT OF INTEREST STATEMENT

The author declares no conflict of interest for this article.

## ETHICS STATEMENT

Approval of the research protocol: N/A.

Informed consent: N/A.

Registry and the registration No. of the study/trial: N/A.

Animal studies: N/A.
